# The Potential of Lactic Acid Bacteria and Dairy By-Products in Controlling *Campylobacter jejuni* in Poultry

**DOI:** 10.3390/microorganisms13050996

**Published:** 2025-04-26

**Authors:** Naga Pavan Kumar Reddy Jonnagiri, Gintare Zakariene, Naila Nawaz, Ausra Gabinaitiene, Artūras Stimbirys

**Affiliations:** Department of Food Safety and Quality, Lithuanian University of Health Sciences, LT-44307 Kaunas, Lithuania; nagajonn0524@kmu.lt (N.P.K.R.J.); naila.nawaz@stud.lsmu.lt (N.N.); ausra.gabinaitiene@lsmu.lt (A.G.); arturas.stimbirys@lsmu.lt (A.S.)

**Keywords:** *Campylobacter jejuni*, *Lactobacillus*, feed additives, food safety

## Abstract

*Campylobacter jejuni* (*C. jejuni*) is the primary *Campylobacter* species and a major cause of foodborne illness associated with poultry products. This review focuses on lactic acid bacteria (LAB), especially *Lactobacillus* species, and acid whey as a dairy by-product for *C. jejuni* control in poultry as a sustainable method. LAB strains *L. crispatus* exhibit a cecal colonization reduction of >90% by competitive exclusion and bacteriocin activity, while *L. johnsonii* FI9785 decrease bacterial load 4–5 log_10_. Acid whey, which is abundant in organic acids (e.g., lactic acid) and bioactive peptides (e.g., lactoferrin), reduces *C. jejuni* viability, decreasing the food product contamination on the carcass for a short time by 40%. LAB antimicrobial function becomes more effective when used with acid whey, although specific farm-related variables require additional optimization. Some of the key strategies include co-encapsulating LAB with acid whey or plant-derived antimicrobials for improving survival, conducting in vivo trials in commercial farm conditions to evaluate scalability, and adding whey into feed (1–2% inclusion) or applying it as a pre-slaughter spray. These strategies enable the antibiotic-free production and circular economy goals through repurposing low-cost acid whey. Future studies should directly compare them with standard antimicrobials to confirm their scalability for poultry safety.

## 1. Introduction

*C. jejuni*, a Gram-negative, thermophilic bacterium, is the most common cause of bacterial foodborne gastroenteritis worldwide, with poultry identified as the primary reservoir for human infection [1]. The European Food Safety Authority (EFSA) considers that 20–30% of human campylobacteriosis cases arise from the mishandling or undercooking of poultry products, while 50–80% are epidemiologically linked to poultry exposure [2]. Contamination associated with cross-contamination during food handling and the consumption of undercooked meat has high potential health and economic impacts on public health [3]. Among *Campylobacter* species, *C. jejuni* and *C. coli* are of most clinical importance, which are found in the gastrointestinal tracts of poultry due to their adaptation to high body temperatures (41–42 °C) [4]. However, other species such as *Campylobacter lari* and *Campylobacter upsaliensis* may occasionally cause illness in humans (Table 1). Their impact is less significant compared to *C. jejuni*, which remains the predominant pathogen in poultry-associated outbreaks [5]. Current antimicrobial methods have severe limitations. Antibiotics like fluoroquinolones are restricted due to rising resistance and bans [6]. Non-antibiotic alternatives like organic acids and vaccines have poor efficacy, are expensive, and lack consumer confidence. These gaps highlight the need for sustainable, social, and cost-effective solutions based on One Health principles [7].

To overcome these limitations, lactic acid bacteria (LAB) have emerged as effective biological tools for reducing *C. jejuni* in poultry. LAB, particularly strains such as *L. crispatus*, *L. salivarius*, *L. helveticus*, and *L. gallinarum*, inhibit *C. jejuni* through bacteriocin production, competitive exclusion, and pH manipulation [8,9]. Studies show that LAB demonstrate effective capabilities in lowering *C. jejuni* growth in the gastrointestinal tracts of poultry, thereby promoting safer meat outcomes [10]. These characteristics make LAB attractive probiotic candidates in the context of sustainable and natural poultry health management. These outcomes comply with the capability of LAB strains in decreasing the levels of *Campylobacter* in poultry production. In addition to LAB, dairy by-products such as whey have shown promising antimicrobial properties against *C. jejuni.* The inclusion of dairy by-products, such as whey, can be considered substrates for producing bioactive compounds that inhibit the growth of *Campylobacter*, while also valorizing waste [11]. Even though poultry are unable to produce the lactase enzyme for lactose digestion, research indicates that fermented or processed dairy by-products overcome such limitations, thus allowing them to be used as feed additives [12].

This review paper examines the effects of lactic acid bacteria (LAB) and dairy products on *C. jejuni* contamination in poultry. It evaluates their possible benefits, mechanism of action, and efficacy for decreasing the pathogen. Furthermore, it assesses the impact of using LAB and dairy products on poultry safety. The analysis focuses on their practical applications as natural interventions in controlling microbial risks.

**Table 1:** Different types of *Campylobacter* Species, Transmission routes, and Human Illness.

Campylobacter Species	Primary Transmission Routes	Clinical Manifestations in Humans
***C. jejuni*** [3,13]	Consumption of undercooked poultry Raw/unpasteurized milk Contaminated water Contact with animals (especially poultry)	Gastroenteritis Abdominal pain and fever Guillain-Barré syndrome
***C. coli*** [14]	Consumption of contaminated pork Poultry products	Gastroenteritis Bacteremia
***C. lari*** [3]	Wild birds and seagulls Contaminated water Shellfish	Gastroenteritis Bacteremia (rare)
***C. upsaliensis*** [3]	Domestic pets (dogs and cats) Person-to-person contact	Gastroenteritis Breast abscess (rare) Bacteremia (rare)

## 2. The Role of *C. jejuni* in Public Health: Prevalence and Impact

### 2.1. Epidemiology and Burden

*C. jejuni* is recognized as the leading bacterial cause of gastroenteritis worldwide [15]. The World Health Organization (WHO) reports that in 2010, about 95 million people became ill from foodborne *Campylobacter*, based on confirmed case analyses and modeling data from surveillance studies, together with systematic review findings and burden of disease assessments [16]. In recent years, reported human *campylobacteriosis* cases have risen, overtaking *Salmonella* infections in many regions. For example, the European Union reported 220,000 cases of *campylobacteriosis* in 2019 as compared to 88,000 cases of *salmonellosis* [17,18].

Poultry is the major reservoir for thermophilic *Campylobacter*, such as *C. jejuni*, *C. coli*, and *C. lari*, with commercial farms showing 50–70% prevalence of *C. jejuni* colonization. Contaminated poultry products are responsible for 20–30% of human infections, primarily through undercooked meat consumption or cross-contamination [19,20].

### 2.2. Clinical Significance

In humans, *C. jejuni* infections vary from mild gastroenteritis (diarrhea, fever, cramps) to severe complications such as Guillain-Barré syndrome (GBS), a serious autoimmune disorder that affects 1–2 cases per 100,000 population per year. Moreover, reactive arthritis and bacteremia are clinically relevant complications, particularly in vulnerable groups such as the elderly and immunocompromised individuals [1]. Rising antimicrobial resistance, notably fluoroquinolone resistance rates of 30% in Asia and 20% in the EU, compounds treatment challenges, underscoring the need for preventive strategies targeting poultry, the primary reservoir [21]. Reducing *C. jejuni* colonization in poultry through feed additives like lactic acid bacteria (LAB) and dairy by-products could directly mitigate these human health risks by disrupting transmission at the source.

### 2.3. Transmission Pathways and Prevention Strategies

Although contaminated poultry is the major source of human *C. jejuni* infection, transmission occurs through multiple pathways. *C. jejuni* is primarily spread through contaminated poultry, with horizontal transmission occurring via fecal shedding within poultry flocks. Fecal ingestion by birds is the main source of spread in poultry farms. Vertical transmission through eggs is minimal [22]. Other transmission routes include contaminated feed, water, insects, and farm equipment. Insects (flies and beetles) can spread *C. jejuni* by means of feces and contaminated water. Rodents and wild animals may also contribute, while farm workers and contaminated equipment play a role in transmitting the bacteria between farms [19,23,24].

The prevention of *C. jejuni* infections needs a multi-faceted approach at different stages throughout the food chain. On poultry farms, biosecurity measures like fecal contamination control and enhanced hygiene procedures are essential [25]. Good sanitation practices and management of the environmental vectors, such as insects and rodents, can reduce the transmission [26]. Feed additives such as lactic acid bacteria (LAB) and dairy by-products have also shown potential for reducing *C. jejuni* colonization in poultry [11,27]. Additionally, ensuring proper cooking and handling of poultry, avoiding cross-contamination, and using safe water sources are crucial to minimizing human exposure. Surveillance and monitoring at all stages, from farm to table, also help in early detection and control. Proper cooking and handling of poultry, prevention of cross-contamination, and ensuring a safe water supply are essential for reducing human infection. Surveillance and monitoring at all points, from farm to table, will also assist in early detection and control [28].

## 3. Evaluation of Different Poultry Feed Additives in the Reduction of *C. jejuni*

The study of poultry feed additives represents a new approach to decreasing the *C. jejuni* population in poultry birds. Recent research examines both natural and synthetic additives for their antimicrobial properties because they could restrict *C. jejuni* growth in the gastrointestinal tract [29,30]. The inclusion of lactic acid and citric acid along with malic acid in poultry feed is a common practice, as these organic acids decrease gut pH, thus preventing pathogen survival [31]. Based on their MIC values, which range from 0.5 mg/mL to 4.1 mg/mL, these organic acids show significant potential for reducing *C. jejuni* growth. This supports their effectiveness at commercial inclusion levels, making them a practical approach for reducing *C. jejuni* in processing [32]. Plant extracts, like thyme and oregano, decrease bacterial numbers and prevent *Campylobacter* multiplication in poultry [33]. It was observed that the combinations of oregano, lactic acid, and sorghum by-product exhibit synergy effects against *C. jejuni*. Oregano essential oil demonstrated the strongest antimicrobial effect, with an MIC of 0.0038%, followed by thyme (MIC: 0.006%), while lactic acid required a higher concentration (MIC: 0.05%). Oregano was more effective than lactic acid in reducing *Campylobacter* growth. The oregano and lactic acid combinations had enhanced antimicrobial activity, with synergy occurring at concentrations such as 0.0005% oregano and 0.015% lactic acid [34].

Essential oils from plants such as garlic and ginger demonstrate bacterial growth inhibition properties for poultry [30]. *C. jejuni* populations decrease when exposed to essential oils from plants because these natural products display antimicrobial properties that block bacterial cellular processes and growth [35]. The method aims to improve food safety through reduced *Campylobacter* quantities in poultry products [36]. Prebiotics, like inulin or fructooligosaccharides, support beneficial gut bacteria, indirectly suppressing *C. jejuni* [37]. It was reported that 1.0% chicory root-derived inulin reduced counts of *Campylobacter* from 5.4 log CFU/g (control) to 3.8 log CFU/g (treated birds) in female birds. Although male broilers exhibited a similar trend, the reduction in *Campylobacter* counts was not statistically significant [38]. Similarly, it was observed that a 2% FOS inclusion in chick diets significantly decreased *C. jejuni* colonization. These results emphasize the potential of prebiotics as a viable approach to control *Campylobacter* in poultry [39].

Moreover, some recent studies have demonstrated the feed application of fatty acids and their antimicrobial properties, especially medium-chain fatty acids (MCFAs). MCFAs such as lauric acid and caprylic acid are known to be effective in lowering *C. jejuni* colonization by interfering with bacterial cell membranes and preventing the ability of bacteria to bind to the gut lining [40]. The use of phytase along with protease improves gut health and phosphorus access, thus creating conditions that limit *C. jejuni* survival in addition to the improved nutrient absorption, which maintains gut well-being [41].

Short-chain fatty acids, including butyrate, acetate, and propionate, provide an antimicrobial effect to *C. jejuni* by interfering with its metabolic functions and decreasing intestinal pH levels. It was found that butyrate (12.5 mM at pH 6.0) had strong bactericidal effects in vitro but failed to reduce colonization in vivo. Acetate and propionate also inhibited growth in vitro, while *L-lactate* showed no effect. In comparison, MCFAs such as caprylic and lauric acids have a stronger ability to break the membranes of bacteria and prevent adhesion [42]. Although butyrate showed bactericidal effects in vitro, it was less effective in vivo. Therefore, MCFAs appear to offer a more reliable and effective solution for reducing *C. jejuni* in poultry [43].

Another method employs the use of bacteriophages directly added to poultry feed as a supplement to decrease the colonization of *C. jejuni*. This study isolated six *C. jejuni*-specific phages with CPS2 among them, which showed high specificity to *C. jejuni* and reduced its colonization. Despite their narrow host range, these phages show promise as a targeted biocontrol strategy for poultry farming, effectively suppressing *C. jejuni* while maintaining beneficial gut microbiota [44].

These feed additives, organic acids, plant extracts, medium-chain fatty acids, short-chain fatty acids, bacteriophages, enzymes, and prebiotics act in distinct mechanisms to reduce *C. jejuni* levels in poultry. Various feed additives composed of organic acids, plant extracts, and medium-chain fatty acids together with enzymes and probiotics aim to decrease the count of *C. jejuni* in poultry. The additives improve gut health and increase the safety of poultry products for consumer consumption.

## 4. Lactobacillus Strains as Probiotic Inventions

Lactic acid bacteria, particularly *Lactobacillus* species, have been regarded as a probiotic in the inhibition of *C. jejuni* colonization in broiler chickens [45]. Animal health and food safety might be controlled using the advantages of the replacement of the antibiotic growth promoters in food-animal production by lactic acid bacteria (LAB) and their bacteriocins, highlighting their potential to enhance animal health and food safety by controlling animal and foodborne pathogens. Their antimicrobial compounds, such as bacteriocins, offer a natural and sustainable alternative to antibiotics, which will help lower the resistance to antibiotics in both animals and humans [46]. To enhance the efficacy of probiotic interventions, strain selection should concentrate on identifying *Lactobacillus* that presents high environmental stress resistance, including tolerance to gastric acidity, temperature variations, and bile salts [47]. These properties are critical for survival throughout the gastrointestinal tract and maintaining antimicrobial activity in vivo.

The effect of prebiotics and feed was also evaluated on the health of chicken and gut microflora, among others, including the enhancement of beneficial bacteria like *Lactobacillus*. For instance, *Lactobacillus crispatus* was reported to decrease *C. jejuni* in the ceca of broilers by competitive inhibitory effect and by the production of lactic acid [48]. It was found that only 4 out of 10 treated chickens were colonized, and none exceeded bacterial loads of 10^5^ CFU/g. These data show two positive outcomes: one is the decreased prevalence of colonization, and the other is the reduction in bacterial load that fits a scenario where *Lactobacillus crispatus* restricts *C. jejuni* establishment within poultry and builds suppression of bacterial growth in those that are colonized [8]. Probiotic treatments with *Lactobacillus* species minimize *C. jejuni* shedding on farms, lowering contamination risks in poultry products [49]. Specific strains like *Lactobacillus johnsonii* FI9785 reduce *C. jejuni* colonization by up to 4–5 log_10_ in poultry [50], aligning with consumer demand for natural, sustainable practices. In vitro, partial inhibition was determined via the Well Diffusion Method, with inhibition zones of 11.0 ± 1.5 mm for *Lactobacillus salivarius* and 12.5 ± 1.8 mm for *Lactobacillus reuteri*. Conversely, the Agar Slab Method inhibited *C. jejuni* and *C. coli* effectively by *Lactobacillus salivarius* and *Lactobacillus reuteri*, with inhibited zones of 21.0 ± 2.0 to 20.3 ± 2.3 mm. A greater inhibition in the Agar Slab Method is probably a result of a longer duration of release of antimicrobial compounds, possibly facilitating the increased interaction with bacteria [51].

*Lactobacillus* strains are capable of causing a significant decrease in the count of *C. jejuni* in poultry by inhibiting the adhesion to the intestinal epithelial cells, which is decisive for colonization [49]. Other lactic acid bacteria such as *Lactobacillus helveticus* (*L. helveticus*) and *Lactobacillus paracasei* show similar potential [52]. It was demonstrated that *Lactobacillus gasseri* LG2055 significantly reduced *C. jejuni* invasion of human intestinal cells by more than 2 log and decreased chick colonization. This inhibition is attributed to acid production and competition for epithelial adhesion sites [53].

Table 2 outlines key strains and their mechanisms and efficacy. *L. helveticus* is not only effective against *C. jejuni* but also improves gut health by elevating beneficial bacteria [54]. The antibacterial properties of *Lactobacillus* species are not the only reason for using them to control the immune system of poultry, but also to contribute to improving the resistance to pathogens and overall health [55]. Furthermore, these probiotics provide a viable alternative to antibiotics, addressing antimicrobial resistance and supporting sustainable poultry production [56]. The antibiotic growth promoters used as feed additives have been banned in the European Union since 2006, and this is contributing to the worry about other means, like probiotics, for maintaining the health and performance of poultry [57].

The collagen-binding protein (*CBP*) of *Lactobacillus fermentum* 3872, which functions as a surface adhesin, effectively inhibits *C. jejuni* binding to collagen receptors, thus blocking its colonization in the host. *Lactobacillus* strains reduce *C. jejuni* quorum-sensing activities by down-regulating virulence genes, including *luxS*, *ciaB*, and *flaA*, and this decreases adhesion, invasion, and motility [58]. Probiotic lactobacilli could increase host immunity by stimulating macrophage activation, nitric oxide secretion, and phagocytosis, which would be beneficial for overall immunity [28]. Regulatory authorities, including *EFSA* and the *FDA*, recognize *Lactobacillus* strains as safe (*GRAS*) for improving poultry health without antimicrobial resistance risks [59,60,61]. For instance, *EFSA* confirmed the safety of *L. acidophilus* D2/CSL and *L. plantarum* DSM 26571 as feed additives [62,63]. Although poultry do not possess lactase enzymes for the digestion of dairy products [12], commercial probiotics like *PoultryStar* (administered via drinking water) reduced *Campylobacter* counts by 1.88 log at day 35. *Ecobiol* (*Bacillus amyloliquefaciens*) also reduced *C. jejuni* level at day 42, although the results were not statistically significant due to high variability [64,65]. When added to feed (1 g/kg), *PoultryStar ME* boosts broiler performance and nutrient digestion while *Ecobiol* (1 kg/ton feed) boosts feed conversion ratio [66,67].

## 5. Acid Whey: A Dairy By-Product with Antimicrobial Potential in *C. jejuni* Control in Poultry

Whey, one of the by-products of cheese and yogurt manufacturing, has become more prominent because of its remarkable antimicrobial action [68]. Whey has anti-bacterial properties, as it is rich in organic compounds, such as lactic acid, acetic acid, and propionic acid; hence, it has the ability to inhibit the growth of many types of Gram-negative bacteria (*C. jejuni*) [69]. These factors are also important in the antimicrobial activity of acid whey, as it decreases the environment’s pH, making it an extremely hostile site for many pathogens [70]. Bioactive peptides such as lactoferrin, found in whey, prevent bacterial cell wall association, nutrient uptake, and the inhibition of *C. jejuni* survival [71]. Hence, these attributes can be considered a viable alternative to synthetic antimicrobials in poultry processing.

The effectiveness of 50% whey might reduce *C. jejuni* on chicken carcasses. The experiment comprised dipping and spraying treatments, and microbial load assessment was performed on days 3, 4, and 7. It was revealed that spraying with whey lowered *C. jejuni* levels at day 3 and day 4, which suggested that whey could be an intervention tool for the control of pathogens in the short term. In contrast, dipping treatments showed no significant reduction of *C. jejuni* survival by day 7 compared to the control group (T1) or synthetic antimicrobial treatments (T2: nisin, T4: N. sativa). This decline in efficacy over time might be due to pH neutralization by cold storage (4 °C) and breakdown of the whey bioactive compounds (e.g., organic acids, lactoferrin), which mainly contribute to antimicrobial activity [72]. To improve the stability of acid whey and retain its antimicrobial activities during storage under cold conditions, it can be encapsulated in protective matrices (alginate or chitosan) or incorporated with stabilizing agents like plant-derived polyphenols. These approaches help preserve its bioactive compounds and sustain low pH levels [73]. However, some research suggests the combination of acid whey with other probiotics or natural antimicrobial compounds to enhance it and widen its potential for use in food safety applications [74].

Acid whey can be applied through several targeted methods to control *C. jejuni* in poultry. Its inclusion in poultry feed has been shown to reduce the *C. jejuni* levels by up to 40% because of its effect on the gut microbiota [75]. Additionally, spraying acid whey onto birds before slaughter may decrease stress-induced pathogen shedding, which helps in lowering microbial loads before processing [76], while pre-carcass dipping directly inactivates the pathogen [77]. These methods are potential alternatives for controlling *C. jejuni* in poultry contamination.

Whey application as a decontamination agent needs to meet the requirements of both food safety regulations (e.g., FDA’s GRAS status or EFSA approval for antimicrobial efficacy) and labeling guidelines [59,60,61]. Environmental regulation, like EU Directive 2020/741, governs waste disposal, and the residue thresholds for whey-derived compounds must adhere to globally established safety standards [78]. Regional regulatory disparities (e.g., EU vs. U.S. frameworks) and mandatory safety data further challenge implementation.

## 6. Challenges in Utilizing Lactic Acid Bacteria and Dairy By-Products for Poultry Safety in *C. jejuni*

The interest in lactic acid bacteria (LAB) together with dairy waste products like whey for controlling *C. jejuni* in poultry has increased due to their antimicrobial properties. Poultry safety using LAB to control *C. jejuni* has some limitations and scope for future research. The primary limitation is with the intricate nutritional needs of LAB, as this will influence the viability and antimicrobial nature of LAB. The organic acids combined with bacteriocins produced by LAB decrease *C. jejuni* counts, but their ability to grow is restricted by bacteriocins, which create unfavorable conditions [79].

The antimicrobial activity, along with the effectiveness of LAB strains, differs significantly because strain selection depends on dosage and application conditions [80]. Research shows that *Lactobacillus salivarius,* together with select other LAB strains can eliminate *C. jejuni* from poultry samples by 99.9%, but these cases are rare. The majority of LAB strains used for analyzing inhibitory effects on *C. jejuni* proved ineffective since inhibitory rates exceeded 96%. The successful production of these new strains needs two steps: first, isolating them and then creating optimal dosage, gut pH, and nutrient conditions for bacteriocin development [81]. The large-scale production of bacteriocin requires complex efforts that combine high expenses with extensive experimental attempts. Additional work must be conducted to optimize LAB strains for improved strain selection protocols and application methods because their effectiveness varies inconsistently between research studies due to variations in strain efficacy and dosages, and gut microbiota interactions [19]. Dairy waste material like whey poses difficulties regarding its impact on sensory characteristics of poultry products [82]. Bioactive peptides present in whey exhibit antimicrobial properties through which they reduce *C. jejuni* bacterial counts [71]. Studies show that adding whey or whey-derived bioactive molecules to poultry products may result in modifications of flavor, together with texture and final consumer satisfaction [83]. The growth and bacteriocin production of lactic acid bacteria in poultry are considered to be limited due to low levels of the sulfur-containing amino acids (e.g., methionine, cysteine) and the B vitamins (e.g., B12, folic acid) that are necessary for growth and the synthesis of antimicrobial peptides. The consumption of these resources by gut microbes also limits LAB function, impeding the functioning of bacteriocin pursuit against pathogens [84]. One of the main challenges in utilizing LAB and acid whey for microbial control is balancing cost-effectiveness with efficacy. Acid whey tends to be the more economical option, especially for large-scale use, as long as it is locally available. However, LAB is the method with more predictable results regarding microbial inhibition, but it is often considered to have a higher production and application cost [85,86]. The implementation scalability of using LAB along with acid whey for *C. jejuni* control in poultry requires economical production procedures followed by regulatory approval that allows for their integration into standard food safety protocols. Acid whey has the benefit of being a low-cost by-product, but the large-scale use of acid whey is dependent on logistical considerations. LAB has strong antimicrobial properties, which demonstrate promising scalability after improving its production methods and application procedures, despite its higher cost compared to whey [87,88].

The use of LAB, dairy waste products like whey, and feed additives shows potential in controlling *C. jejuni* in poultry. Research requiring additional focus needs to explore LAB nutritional needs and strain differences while studying the sensory consequences of adding LAB to poultry products. Researchers should focus their future work toward optimizing LAB strains while solving these obstacles to enhance poultry safety outcome.

## 7. Future Directions in Utilizing Lactic Acid and Dairy By-Products

Despite these challenges, potential areas exist to enhance the effectiveness of LAB and dairy by-products as *C. jejuni* control agents in poultry. Manufacturing LAB strains resistant to poultry processing environments will ensure their success in future applications [89]. The interest in growing poultry food industry in advanced microbial control technology was explained by showing how this technology enhances food safety and shelf life while reducing conventional preservatives in food. In their review, they investigate multiple preservation strategies that include natural antimicrobials together with packaging solutions and new preservation approaches. Future studies should optimize LAB biocontrol efficacy by combining them with other natural antimicrobial compounds [90].

A combination of *L. Salivarius 1234C*, *L. Agilis 1235C*, and *E. Durans 12311C* LAB strains with plant-derived antimicrobials and essential oils or bacteriocins could lead to the generation of synergistic effects that will improve pathogen inhibition and gut health while enhancing poultry performance. Through this technique, researchers have the potential to develop sustainable probiotic chicken products that improve the efficiency of intensive poultry farming practices [91]. Research studies show that linking LAB with different natural antimicrobial substances leads to elevated microbial activity, thereby developing expanded methods for poultry pathogen control [92]. Such a combination approach would be effective in treating the problem of LAB strain diversity by offering additional modes of action, affecting *C. jejuni* from multiple different angles.

Future work should also develop flavor and texture profiles to mask off-tastes while conducting market research to boost consumer acceptance and sustainable marketing strategies [93]. The research on dairy by-products for poultry safety comprises the development of integrated waste treatment systems that integrate bioenergy recovery and microbial degradation to decrease waste and improve pathogen control. Advances in microorganism research, organic acids, nanoparticles, and genetically modified organisms may further improve poultry safety [94]. The application of bioactive peptides from whey protein is likely to further improve poultry safety by offering antimicrobial and health-related benefits. Further research should search for better fermentation process realization and the selection of lactic acid bacteria strains for increasing bioactive properties of whey-derived additives for poultry [95].

The co-encapsulation of LAB with acid whey proteins or plant-based antimicrobials may enhance delivery and stability, allowing for multi-targeted *C. jejuni* inhibition [96]. For example, the combination of *sophorolipids* with lactic acid decreased the amount of *C. jejuni* in poultry and produced additive effects as a natural sanitizer [97]. Also, the combination of LAB with bacteriocins (e.g., *Lacticin 3147*) and high-pressure processing increases the antimicrobial activities against the pathogen *C. jejuni*. Encapsulation technologies (e.g., spray drying, biofilm-based carriers) are needed to improve the survival of LAB during storage and transit through the gastrointestinal tract [98]. Application methods such as feed supplementation to promote gut colonization, surface spraying to reduce post-slaughter contamination, and marination to combine antimicrobial effects with a shelf life enhancement [99]. In vivo poultry studies under farm conditions are required to confirm the validity of LAB efficacy on reducing *C. jejuni* colonization and to find out if there is synergism between LAB and dairy by-products like acid whey, which can be used both as a nutrient-rich carrier and a prebiotic enhancer [45,100].

## 8. Conclusions

The increasing prevalence of *C. jejuni* in poultry ensures that this foodborne pathogen is a major global public health concern, being responsible for a significant number of foodborne infections across the world. As it can survive in poultry without causing disease, preventing its transmission to humans is necessary. As an example, when it comes to LAB strains like *Lactobacillus crispatus*, research found that they are able to reduce cecal colonization by >90% and *L. johnsonii* FI9785 reductions of 4–5 log_10_, equivalent to traditional antibiotics without contributing to resistance. Conventional practices, for example, the use of antibiotics, pose a problem such as antimicrobial resistance, and there is a requirement for natural and sustainable options.

Lactic acid bacteria (LAB), especially *Lactobacillus*, have been recognized as possible probiotic agents because of their ability to inhibit *C. jejuni*. LAB can decrease pathogens via competitive exclusion, lactic acid production, and bacteriocin secretion, and consequently, they are effective natural alternatives. Acid whey, a dairy processing by-product, also shows a novel antimicrobial solution to expand the reduction of *C. jejuni* contamination. Short-term application of acid whey decreases carcass contamination by 40% while its bioactive peptides (e.g., lactoferrin) disrupt the adhesion of bacteria. In all, the supplementation of LAB as well as the use of acid whey for natural antimicrobial agents is the most promising toward reducing *C. jejuni* contamination in poultry. The evaluation of poultry feed additives together with LAB and acid whey represents a promising research direction. Co-encapsulation of LAB with acid whey or plant-based materials (e.g., oregano oil) may increase stability and efficiency under farm conditions. The literature indicates that organic acids combined with probiotics and plant extracts are natural additives that can potentially reduce the ability of *C. jejuni* to colonize poultry farms.

Research advances in this area will help the poultry industry progress toward sustainability, a transition to antibiotic-free production, the improvement of animal and human health, and addressing global food safety concerns. The implementation of large in vivo trials on commercial farms will overcome the pre-requisite cost-effectiveness (e.g., acid whey decreases waste disposal costs by 20–30%) and the scalability of these solutions.

## Figures and Tables

**Table 2:** *Lactobacillus* Strains as Probiotic Interventions Against *Campylobacter* in Poultry.

Lactobacillus Strain	Mechanism of Action Against Campylobacter	Effectiveness in Poultry
***L. crispatus*** [8,48]	Competitive exclusion Production of lactic acid	Reduced *C. jejuni* colonization in the ceca of broilers Decreased shedding of *C. jejuni*
***L. salivarius*** [44,54]	Production of antimicrobial compounds Competitive exclusion	Enhanced growth performance of white leghorn chickens Reduced *C. jejuni* colonization
***L. helveticus*** [51,52]	Production of bacteriocins Improvement of gut health Elevation of beneficial bacteria levels	Significant reduction in *Campylobacter* colonization Improved overall gut health of poultry
***L. paracasei*** [51,54]	Production of antimicrobial compounds Competitive exclusion Modulation of intestinal microbiota	Prevention of *C. jejuni* colonization in poultry Improved resistance to pathogens

## Data Availability

No new data were created or analyzed in this study.
